# Rabbit aneurysm models mimic histologic wall types identified in human intracranial aneurysms

**DOI:** 10.1136/neurintsurg-2017-013264

**Published:** 2017-08-02

**Authors:** Shunli Wang, Daying Dai, Praveen Kolumam Parameswaran, Ramanathan Kadirvel, Yong-Hong Ding, Anne M Robertson, David F Kallmes

**Affiliations:** 1 Department of Radiology, Mayo Clinic, Rochester, Minnesota, USA; 2 Department of Pathology, Shanghai East Hospital, Tongji University, Shanghai, China; 3 Center for Biological Imaging, University of Pittsburgh, Pittsburgh, USA

**Keywords:** Intracranial aneurysms, animal model, histological subtypes

## Abstract

**Background:**

Semiquantitative scales correlate histopathologic findings in the walls of human aneurysms with rupture status.

**Objective:**

To apply a semiquantitative scale to the rabbit elastase-induced aneurysm model to determine whether rabbit histologic types mimic the full range of histologic subtypes of humans.

**Materials and methods:**

Twenty-seven elastase-induced female rabbit aneurysms were studied, harvested at 2 weeks (n=5) and 12 weeks (n=22). Paraffin-embedded sections received hematoxylin-eosin and Verhoeff-Van Gieson staining. Immunohistochemistry was performed for α-smooth muscle actin and CD31 for endothelial cells. A semiquantitative scale was used for scoring based on human aneurysm tissue, divided into four subtypes according to cellular and extracellular matrix findings: type A, linear organized smooth muscle cells (SMCs) and intact endothelium; type B, thickened wall with disorganized, proliferating SMCs; type C, thick, collagenized and hypocellular wall with or without organizing thrombosis, and type D, extremely thin, hypocellular wall. Separate scoring was performed of the aneurysm neck and proximal and distal zones.

**Results:**

Findings compatible with all subtypes of human aneurysm tissue were identified. Types A and C were found in 13 (48%) and 11 (41%) of 27 aneurysms and in the proximal and distal wall at both time points. Type B was found in 16 aneurysms (59%), exclusively at the neck at both time points; type D, in 14 aneurysms (52%), exclusively at proximal and distal zones of 12-week aneurysms.

**Conclusions:**

The wall of elastase-induced rabbit aneurysm demonstrates histologic findings similar to the four categories of human cerebral aneurysms based on cellular and extracellular wall content.

## Introduction

The elastase-induced rabbit aneurysm has been widely used to test new generations of devices^1–7^ and to study the hemodynamic milieu of saccular aneurysms.^8–12^ Previous studies^12–14^ showed that this model has geometry similar to that of human cerebral aneurysms and has tissue response similar to humans, following platinum coil embolization.^13^ However, few previous reports have directly compared rabbit and human histologic factors.

Multiple previous studies have given details of histopathologic findings in human aneurysms, many focused on correlating these findings with rupture status. Frösen *et al* 15 analyzed and characterized the cellular milieu in 66 human saccular cerebral aneurysms. Using a four-point categorical scale, they showed that certain types of histologic findings in human aneurysms were associated with rupture status. In this study, we retrospectively analyzed the detailed histopathologic evaluations in 27 elastase-induced aneurysms in rabbits and compared these findings with human cerebral aneurysms as detailed by Frösen *et al*.^15 16^ We aimed to determine whether rabbit histologic characteristics mimic the full range of the findings in humans.

## Materials and methods

### Aneurysm samples

Twenty-seven elastase-induced female rabbit aneurysms were used for this retrospective study. The aneurysm creation procedure has been described previously.^17^ All animal procedures were approved by the Institutional Animal Care and Use Committee at our organization. After the animals were euthanized, aneurysm samples were harvested at 2 weeks (n=5) and 12 weeks (n=22). The 27 harvested samples were fixed in 10% neutral buffered formalin and underwent regular tissue processing, embedded in paraffin. The paraffin blocks were sectioned at 4 µm in a coronal orientation, permitting long-axis sectioning of the aneurysm dome, neck, and parent arteries.

### Histologic analysis and immunohistochemistry

For standard histologic evaluation, the sections were stained with hematoxylin-eosin (H&E) and Verhoeff van Gieson. For immunohistochemistry, sections were prepared as previously described.^18^ Briefly, sections were pretreated with 0.1 mol/L citric acid buffer and microwaved for 15 min. They were incubated in hydrogen peroxide (0.3% in distilled water; 20 min), followed by incubation with normal 5% horse serum (20 min; 37°C), and then with primary antibody (α-smooth muscle actin, Dako; CD31, Dako) at 37°C for 1 hour. Next, they were incubated with primary antibody at 4°C overnight. Slides were rinsed in phosphate-buffered saline (PBS) and incubated with biotinylated secondary horse antimouse immunoglobulin G (Vector Laboratories). Sections were rinsed in PBS and incubated with Vectastain Elite ABC Reagent (Vector Laboratories) for 45 min at 37°C. Finally, slides were developed with diaminobenzidine-tetrahydrochloride (Vector Laboratories). Negative controls were performed with non-immune, normal serum versus the primary antibody.

### Histologic subtyping

Two trained pathologists (SW and DD) with more than 10 years’ experience interpreted the stained sections and classified the aneurysm wall structure. This wall was compared with human aneurysm wall and the classification reported by Frösen *et al*.^15 16^ We segregated the investigated aneurysmal wall into three regions: neck area at and near the interface between parent artery and aneurysm wall; proximal wall, defined as the wall along the aneurysm’s proximal aspect; and distal wall, defined as the wall along the aneurysm’s distal aspect. Scoring was performed using a semiquantitative scale and four distinct subtypes based on cellular and extracellular matrix findings. Subtypes were A, endothelialized wall with linearly organized smooth muscle cells (SMCs); B, thickened wall with disorganized SMCs; C, thick, collagenized and hypocellular wall with or without organizing thrombosis; and D, extremely thin, hypocellular wall. A single aneurysm region (neck and proximal and distal walls) may manifest multiple subtypes.

## Results

### Conventional histopathologic and immunohistochemistry findings

At 2 weeks, the elastic lamina was completely or almost completely degraded within the aneurysmal walls. Distribution of endothelial cells was variable across the sac. In some regions, endothelial coverage was discontinuous or completely absent, whereas other areas appeared normal. A thickened wall with proliferating smooth muscle-like cells or neointimal hyperplasia was observed in large regions of the wall for all aneurysms at this time. Neointimal hyperplasia was found primarily at the transition zone from the parent artery to the proximal part of the aneurysm wall. While some regions were hypocellular, the wall was not thin. Acute inflammatory cell infiltration was seen in one of five aneurysms; inflammatory cells were absent in the other four aneurysms.

At 12 weeks, the elastic lamina was completely degraded in all specimens. Although the neck region still appeared thickened with a proliferation of smooth muscle-like cells, the remainder of the sac was largely replaced with collagenized tissue, devoid of cellular elements. α-Smooth muscle actin-positive cells were barely detected. No inflammatory infiltrate was found within the aneurysmal walls at this time. A laminated, organized thrombus was found at the apices of the aneurysm dome in two of the 23 samples.

### Histologic subtypes

**Figure 1:**
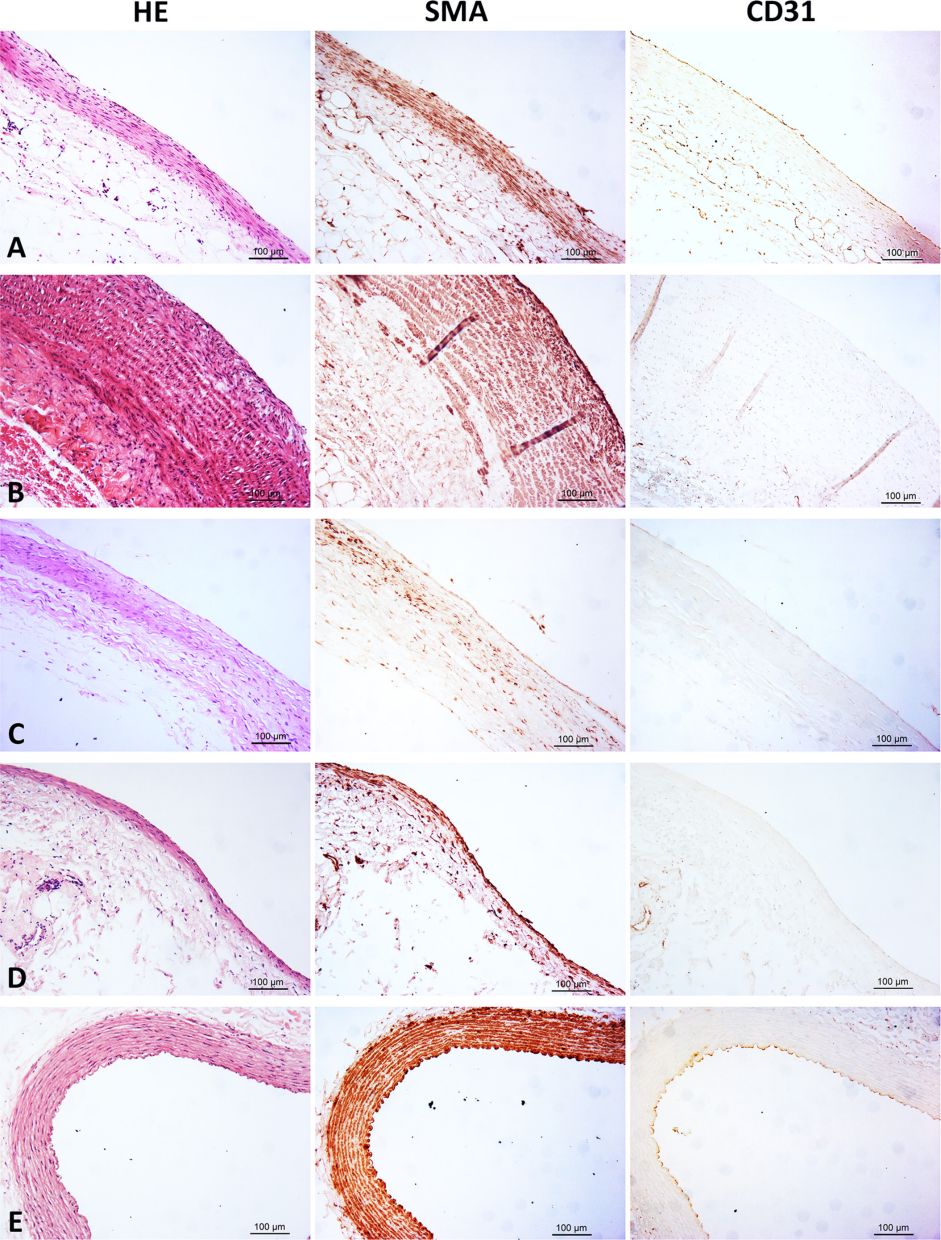
Representative Images of the four subtypes of human intracranial aneurysms identified in the rabbit model (first column: HE, hematoxylin & eosin (H&E); middle column: immunohistochemistry (IHC) for α-smooth muscle actin (SMA); right column: IHC for CD31; original magnification 200× for all photos). (A) Type A: linear organized smooth muscle cell (SMC) and intact endothelium; the lumen side of the wall is still lined with CD31 positive cells. (B) Type B: thickened wall with disorganized, proliferating SMC positive cells; the wall lacks CD31 positive cells coverage. (C) Type C: thick, collagenized, hypocellular wall with/without CD31 positive cells coverage. (D) Type D: extremely thin and decellularized wall. (E) Normal right common carotid artery wall of rabbit. The media of the wall is composed of 11–12 layers of well-organized SMA positive cells, and it is lined with intact CD31 positive cells.

**Figure 2:**
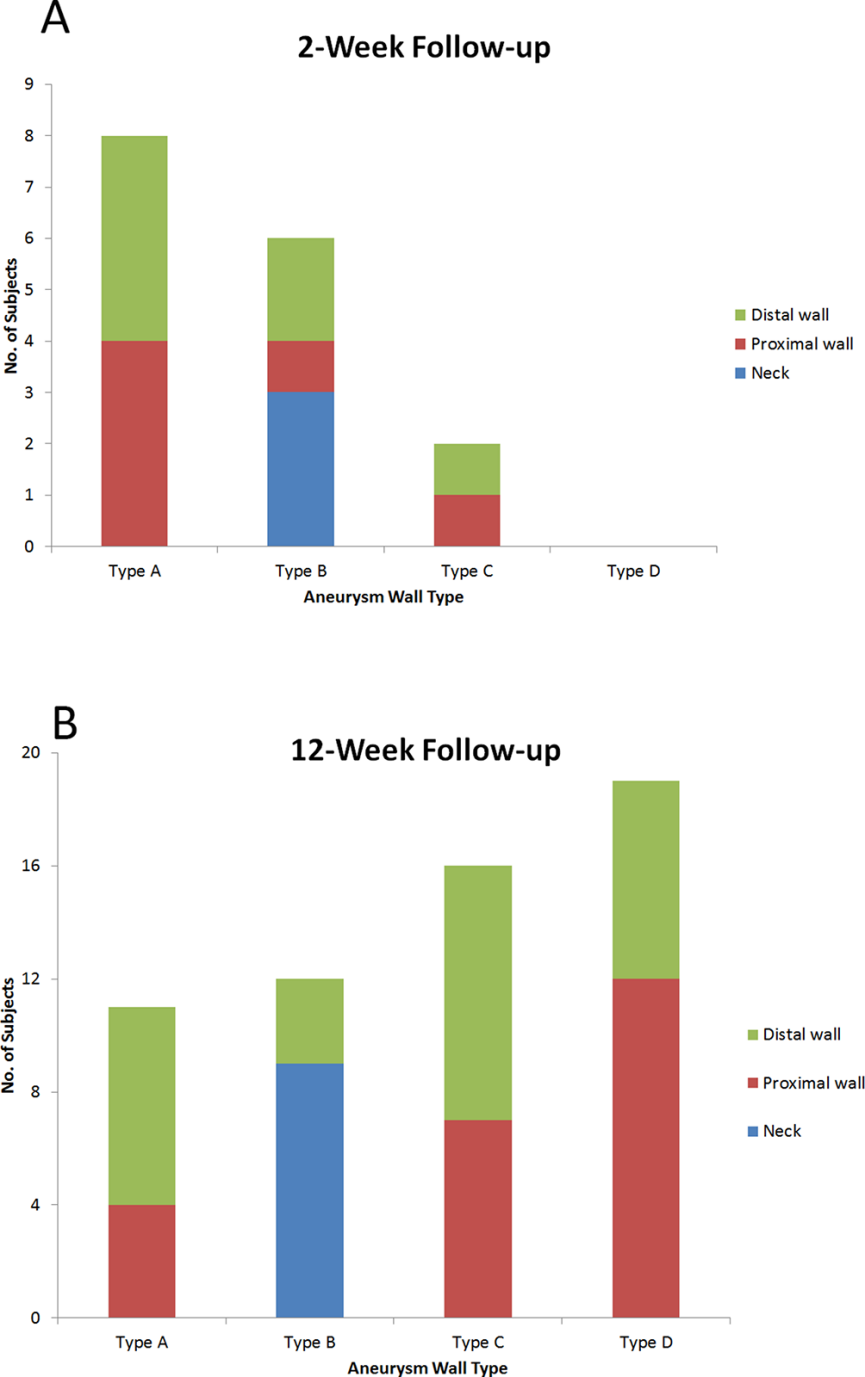
Rabbit aneurysm sacs that display each human aneurysm wall subtype by sac region. (A) At 2-week follow-up. (B) At 12-week follow-up.

Findings compatible with all four subtypes of human aneurysm tissue were identified (figure 1). Overall, types A and C were found in 13 (48%) and 11 (41%) of 27 aneurysms and were present in the proximal and distal walls at both time points (figure 2). Type B was found in 16 aneurysms (59%), exclusively at the neck at both time points. Type D was found in 14 aneurysms (52%), exclusively at the proximal and distal walls of 12-week subjects.

**Figure 3:**
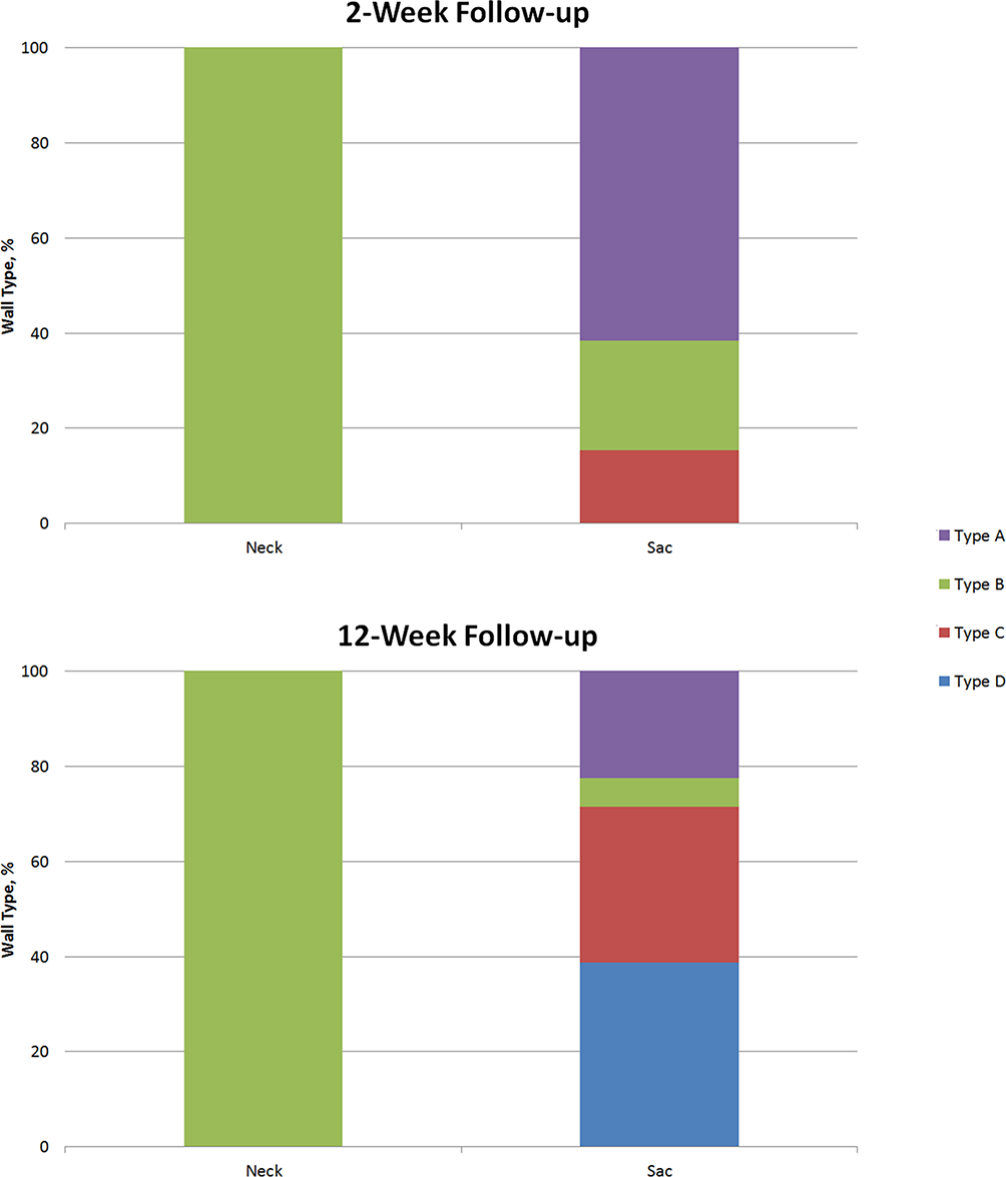
Percentages of aneurysm wall subtypes at 2- and 12-week follow-up by neck and sac region.

In figure 3, distribution of wall types within the neck and sac is considered for the two time points. By 2 weeks, the neck was solely type B and was unchanged at 12 weeks. In contrast, the sac was heterogeneous at both time points and displayed a shifting wall type from more than 80% of types A and B at 2 weeks to more than 70% of types C and D at 12 weeks.

## Discussion

This study demonstrates that categories of histologic change seen in human aneurysm tissue are mimicked in the elastase-induced aneurysm model in rabbits. Each of the four primary categories described by Frösen *et al* 15 16 was seen in the rabbit samples. We noted substantial variation in subtypes according to the region of the aneurysm wall and also over time. Types A, B, and C were present at both time points. These three types are associated with relatively lower rates of rupture at presentation than type D in clinical specimens. The neck region exclusively had type B, characterized by neointima hyperplasia. Of note, type D—universally associated with rupture in clinical specimens—was seen in the vast majority of rabbit samples at 12 weeks but was absent in the 2-week samples.

Lack of an intact elastic lamina is a common finding for human cerebral aneurysms.^15 19–21^ Consistent with these results, the rabbit model shows disruption or some loss of elastic lamina at 2 weeks and complete loss at 12 weeks. Loss of endothelium, reported in 50% of human intracranial aneurysms^15^ and believed to be important in aneurysm wall degeneration,^15^ was found in the rabbit wall types.

Although wall heterogeneity was not the focus of their work, Frösen *et al* 15 noted that several aneurysm walls showed gradual change from type A or B to type C or D, mostly in the direction of neck to fundus. Obtaining entire human aneurysm sacs for analysis is difficult, yet Tulamo *et al* 22 reported one case where they found a progressive change from type B (neck) to type C to type D. These findings are consistent with our 12-week results that showed progression of type B at the neck largely to types C and D in the proximal and distal walls.

Previous studies have noted histologic changes in the rabbit elastase-induced model.^23 24^ Compatible with our current findings, the prior studies noted thin, hypocellular aneurysm walls. This study extends previous studies by using a categorical scale based on categories of human aneurysm specimens shown to correlate with the proportion of ruptured versus unruptured status at presentation.^15^

The rabbit model offers some advantages over clinical tissue. The entire aneurysm sac can be evaluated in rabbits whereas human tissue, when excised during aneurysm surgery, usually consists of small segments of the aneurysm dome. Further, the rabbit model can be used to explore progression of the aneurysm wall at multiple time points. Frösen *et al* 15 conjectured that the histologic wall types identified for humans reflect progressive wall degeneration. This conjecture, which cannot be studied directly in human patients, is supported by the changes seen in the rabbit model. Herein, we describe a progressive shift from a wall dominated by types A and B at 2 weeks to predominantly types C and D at 12 weeks. Progressive changes in the aneurysm wall are the focus of ongoing investigation using the rabbit model.^25^ Together, these findings suggest that the rabbit model might be useful for testing systemic or local therapies aimed at modulating histologic progression to subtypes associated with rupture.

The pathophysiology of human intracranial aneurysms is complex and is related to genetic background and previous disease history, such as hypertension, cholesterol deposit, wall inflammation, and lifestyle—for example, smoking.^26–28^ On the contrary, the elastase-induced rabbit model reported in this study does not have these factors mentioned above, which is a limitation of all animal models including the one used here. However, our previous studies demonstrated that the elastase-induced saccular aneurysm in rabbit is similar to human intracranial saccular aneurysms geometrically and hemodynamically for a number of reasons, including (1) location along a curved vessel with anatomy that simulates human aneurysms such as those in the ophthalmic region^14^; (2) aneurysm size similar to the mean size of human cerebral aneurysms^29^; (3) long-term patency^30^; (4) shares some molecular features with human intracranial aneurysms^31^; (5) hemodynamic and geometric features are qualitatively and quantitatively similar to those seen in large numbers of human cerebral aneurysms.^12^ In addition, the healing process in the rabbit model after embolization with platinum coils mimics the healing seen in human aneurysms.^13^ Because the aneurysm model does not undergo spontaneous rupture, we are limited to analyses of changes over time rather than correlating findings with rupture risk. Further, lack of full knowledge of the location of human aneurysm tissue relative to the aneurysm neck versus dome limits direct comparison between the two species. Organizing thrombus was reported in the lumen of 21% of unruptured and 60% of ruptured aneurysms.^15^ However, it was identified only in the dome of some of the rabbit aneurysms. Finally, because the apex of the rabbit aneurysm dome consists of organizing thrombus, based on the mode of construction with distal ligation, we are unable to evaluate histologic changes high in the aneurysm cavity.

## Conclusions

The wall of the elastase-induced rabbit aneurysm model reproduces the histologic categories found in human aneurysms and offers an opportunity for studying progressive changes in the aneurysm wall.

## References

[1] Dai D, Ding YH, Danielson MA, et al Endovascular treatment of experimental aneurysms with use of fibroblast transfected with replication-deficient adenovirus containing bone morphogenetic protein-13 gene. AJNR Am J Neuroradiol 2008;29:739–44. 10.3174/ajnr.A0892 18184848 PMC2605703

[2] de Gast AN, Altes TA, Marx WF, et al Transforming growth factor beta-coated platinum coils for endovascular treatment of aneurysms: an animal study. Neurosurgery 2001;49:690–4. discussion 94-6. 11523681 10.1097/00006123-200109000-00030

[3] Ding YH, Dai D, Lewis DA, et al Angiographic and histologic analysis of experimental aneurysms embolized with platinum coils, Matrix, and HydroCoil. AJNR Am J Neuroradiol 2005;26:1757–63. 16091526 PMC7975154

[4] Kallmes DF, Ding YH, Dai D, et al A new endoluminal, flow-disrupting device for treatment of saccular aneurysms. Stroke 2007;38:2346–52. 10.1161/STROKEAHA.106.479576 17615366

[5] Kallmes DF, Ding YH, Dai D, et al A second-generation, endoluminal, flow-disrupting device for treatment of saccular aneurysms. AJNR Am J Neuroradiol 2009;30:1153–8. 10.3174/ajnr.A1530 19369609 PMC7051356

[6] Kallmes DF, Fujiwara NH, Yuen D, et al A collagen-based coil for embolization of saccular aneurysms in a New Zealand White rabbit model. AJNR Am J Neuroradiol 2003;24:591–6. 12695186 PMC8148684

[7] Killer M, Kallmes DF, McCoy MR, et al Angiographic and histologic comparison of experimental aneurysms embolized with hydrogel filaments. AJNR Am J Neuroradiol 2009;30:1488–95. 10.3174/ajnr.A1649 19474120 PMC7051620

[8] Kadirvel R, Ding YH, Dai D, et al The influence of hemodynamic forces on biomarkers in the walls of elastase-induced aneurysms in rabbits. Neuroradiology 2007;49:1041–53. 10.1007/s00234-007-0295-0 17882410

[9] Zeng Z, Durka MJ, Kallmes DF, et al Can aspect ratio be used to categorize intra-aneurysmal hemodynamics?--A study of elastase induced aneurysms in rabbit. J Biomech 2011;44:2809–16. 10.1016/j.jbiomech.2011.08.002 21925661 PMC3230080

[10] Zeng Z, Kallmes DF, Ding Y, et al; Hemodynamics of elastase induced aneurysms in rabbut- a new high flow bifurcation model: Proceedings of the ASME 2011 Summer Bioengineering Conference, 2011:SBC2011–53819.

[11] Zeng Z, Kallmes DF, Durka MJ, et al Sensitivity of CFD based hemodynamic results in rabbit aneurysm models to idealizations in surrounding vasculature. J Biomech Eng 2010;132:091009 10.1115/1.4001311 20815643 PMC2936725

[12] Zeng Z, Kallmes DF, Durka MJ, et al Hemodynamics and anatomy of elastase-induced rabbit aneurysm models: similarity to human cerebral aneurysms? AJNR Am J Neuroradiol 2011;32:595–601. 10.3174/ajnr.A2324 21273353 PMC3920548

[13] Dai D, Ding YH, Danielson MA, et al Histopathologic and immunohistochemical comparison of human, rabbit, and swine aneurysms embolized with platinum coils. AJNR Am J Neuroradiol 2005;26:2560–8. 16286401 PMC7976186

[14] Short JG, Fujiwara NH, Marx WF, et al Elastase-Induced saccular aneurysms in rabbits: comparison of geometric features with those of human aneurysms. Am J Neuroradiol 2001;22:1833–7. 11733310 PMC7973827

[15] Frösen J, Piippo A, Paetau A, et al Remodeling of saccular cerebral artery aneurysm wall is associated with rupture: histological analysis of 254 unruptured and 42 ruptured cases. Stroke 2004;35:2287–93. 15322297 10.1161/01.STR.0000140636.30204.da

[16] Frösen J, Piippo A, Paetau A, et al Growth factor receptor expression and remodeling of saccular cerebral artery aneurysm walls: implications for biological therapy preventing rupture. Neurosurgery 2006;58:534–41. discussion 34-41. doi 10.1227/01.NEU.0000197332.55054.C8 16528195

[17] Altes TA, Cloft HJ, Short JG, et al 1999 ARRS Executive Council award. creation of saccular aneurysms in the rabbit: a model suitable for testing endovascular devices. American Roentgen Ray Society. AJR Am J Roentgenol 2000;174:349–54. 10.2214/ajr.174.2.1740349 10658703

[18] Dai D, Ding YH, Danielson MA, et al Modified histologic technique for processing metallic coil-bearing tissue. AJNR Am J Neuroradiol 2005;26:1932–6. 16155137 PMC8148861

[19] Scott S, Ferguson GG, Roach MR Comparison of the elastic properties of human intracranial arteries and aneurysms. Can J Physiol Pharmacol 1972;50:328–32. 10.1139/y72-049 5038350

[20] Tada Y, Austin G1 FS, Dickson D, et al The significance of the extracellular matrix in intracranial aneurysms. Ann Clin Lab Sci 1993;23. 7681275

[21] Robertson AM, Watton PN Computational fluid dynamics in aneurysm research: critical reflections, future directions. AJNR Am J Neuroradiol 2012;33:992–5. 10.3174/ajnr.A3192 22653325 PMC8013264

[22] Tulamo R, Frösen J, Hernesniemi J, et al Inflammatory changes in the aneurysm wall: a review. J Neurointerv Surg 2010;2:120–30. 10.1136/jnis.2009.002055 21990591

[23] Bouzeghrane F, Naggara O, Kallmes DF, et al In vivo experimental intracranial aneurysm models: a systematic review. AJNR Am J Neuroradiol 2010;31:418–23. 10.3174/ajnr.A1853 19875466 PMC7963965

[24] Cesar L, Miskolczi L, Lieber BB, et al Neurological deficits associated with the elastase-induced aneurysm model in rabbits. Neurol Res 2009;31:414–9. 10.1179/174313208X346918 18826754 PMC2694846

[25] Sang C, Kallmes DF, Kadirvel R, et al 5th International Conference on Computational and Mathematical Biomedical Engineering–CMBE2017. 2, 2017:1300–3.

[26] Fan J, Sun W, Lin M, et al Genetic association study identifies a functional CNV in the WWOX gene contributes to the risk of intracranial aneurysms. Oncotarget 2016;7:16104–11. 10.18632/oncotarget.7546 26910372 PMC4941300

[27] Keedy A An overview of intracranial aneurysms. Mcgill J Med 2006;9:141–6. 18523626 PMC2323531

[28] Qureshi AI, Suarez JI, Parekh PD, et al Risk factors for multiple intracranial aneurysms. Neurosurgery 1998;43:22–6. discussion 26-7 10.1097/00006123-199807000-00013 9657184

[29] Short JG, Fujiwara NH, Marx WF, et al Elastase-induced saccular aneurysms in rabbits: comparison of geometric features with those of human aneurysms. AJNR Am J Neuroradiol 2001;22:1833–7. 11733310 PMC7973827

[30] Ding YH, Dai D, Lewis DA, et al Long-term patency of elastase-induced aneurysm model in rabbits. AJNR Am J Neuroradiol 2006;27:139–41. 16418373 PMC7976068

[31] Mangrum WI, Farassati F, Kadirvel R, et al mRNA expression in rabbit experimental aneurysms: a study using gene chip microarrays. AJNR Am J Neuroradiol 2007;28:864–9. 17494658 PMC8134323

